# The Skin-Lightening Power of Tirbanibulin 1% Ointment

**DOI:** 10.1007/s13555-024-01310-0

**Published:** 2024-11-30

**Authors:** Federica Li Pomi, Andrea d’Aloja, Michelangelo Rottura, Mario Vaccaro, Francesco Borgia

**Affiliations:** 1 Department of Precision Medicine in Medical, Surgical and Critical Care (Me.Pre.C.C.), University of Palermo, 90127 Palermo, Italy; 2 Department of Clinical and Experimental Medicine, Section of Dermatology, University of Messina, Messina, Italy; 3 Department of Clinical and Experimental Medicine, Section of Pharmacology, University of Messina, 98125 Messina, Italy

**Keywords:** Actinic keratosis, Lentigines, Skin aging, Solar lentigines, Solar lentigo, Tirbanibulin, Tirbanibulin ointment, Treatment

## Abstract

**Background:**

Tirbanibulin 1% ointment has been licensed to treat non-hyperkeratotic actinic keratosis (AK) on the face and scalp in adults. Recent evidence suggests that, besides the antineoplastic effect, tirbanibulin may also confer substantial cosmetic benefits to patients.

**Methods:**

We report a single-center retrospective study of patients affected by solar lentigines (SLs) and AKs in the context of field cancerization treated with tirbanibulin 1% ointment.

**Results:**

Among 42 patients, 35% (*n* = 15) experienced complete clearance of SLs, while partial clearance was observed in 50% (*n* = 21) of patients. Regarding AKs, complete and partial clearance were observed in 52% (*n* = 22) and 40% (*n* = 17) of patients, respectively. Major study limitations are the small sample size and the absence of a control group.

**Conclusions:**

Our results suggest that tirbanibulin 1% ointment may offer the dual benefit of treating AKs while simultaneously lightening aesthetically bothersome and difficult-to-treat lesions like SLs with just 5 days of application.

## Key Summary Points

**Table:** 

Topical treatments for actinic keratosis (AK) may confer cosmetic benefits to patients, but the skin-lightening effects on solar lentigo (SL) have not yet been reported.
Tirbanibulin 1% ointment has been licensed to treat non-hyperkeratotic AKs on the face and scalp in adults, and excellent patient compliance has been observed due to its mild side effects and short application time of only 5 consecutive days.
We report a retrospective study of 42 patients seeking treatment for AKs in the context of photodamaged areas in which the application of tirbanibulin 1% ointment induced, besides the clearance of AKs, skin-lightening effects on SLs.
Besides excellent AK response at the 57-day follow-up, a complete clearance SL rate of 35% and a partial SL clearance rate of 50% were observed.
Tirbanibulin treatment should be offered to patients affected by both AKs and SLs.

## Introduction

Actinic keratosis (AK) is a common cutaneous intraepithelial neoplasm characterized by the proliferation of atypical keratinocytes in sun-exposed areas, particularly in older individuals with the fair skin phototype. If left untreated, AK lesions may progress to cutaneous squamous cell carcinoma (cSCC), highlighting the need for effective therapeutic interventions [1]. Tirbanibulin 1% ointment (Klisyri^®^, Almirall, S.A., Barcelona, Spain) has been licensed to treat non-hyperkeratotic AK lesions on the face and scalp in adults, with good results and excellent patient tolerability due to its mild side effects and short application time of 5 consecutive days [2]. Its mechanism of action involves the disruption of microtubule function, which leads to the inhibition of cell proliferation and apoptosis induction in dysplastic keratinocytes [3]. In addition to its well-documented efficacy in treating AK lesions in both clinical trials and real-life settings, recent investigations have suggested potential additional cutaneous benefits of tirbanibulin ointment on skin aging [4, 5]. In particular, in our case series on 7 patients, tirbanibulin displayed efficacy for AKs and solar lentigines (SLs), exerting an overall good skin-lightening effect [5]. Also known as sunspots or age spots, SLs are benign hyperpigmented lesions that result from chronic ultraviolet (UV) exposure and represent a hallmark of photoaging; they are usually found in the context of field cancerization and AKs [6]. In order to investigate its efficacy and safety profile for SLs, we performed a study on patients affected by both SLs and AKs treated with topical tirbanibulin.

## Methods

A retrospective single-center evaluation was carried out at the Dermatology Unit of the University of Messina, Italy, between February 2023 and March 2024. Adult patients affected by both AKs and SLs on chronically photodamaged areas of the scalp and face treated with tirbanibulin 1% ointment were included. Exclusion criteria were pregnancy and lactation, known allergies to any component of the study drug, a history of photosensitivity, active infections, immunosuppression, a history of invasive cSCC, Bowen’s disease, basal cell carcinoma, other malignant tumors in the treatment field, and prior therapies with other topical treatments for AKs and photoaging in the last 12 weeks. The procedures followed here were in accordance with the Helsinki Declaration of 1964 and its later amendments and were approved by the local ethical committee. Written informed consent was obtained from all patients for their photographs and medical information to be published.

### Objectives

The study aimed (a) to evaluate the clearance rate of both SLs and AKs at the 57-day follow-up (T1); (b) to assess the potential correlation between the response to SLs and AKs; and (c) to assess the response durability of SLs and AKs in patients with complete clearance at the 6-month follow-up (T2).

### Baseline Evaluation

At baseline (T0), all patients underwent physical examination with the acquisition of clinical images. Clinically collected data included age, gender, and skin phototype. Solar lentigo (SL) and AK locations (face or scalp) were collected as well.

### Treatment Received by Patients

Patients were instructed to apply tirbanibulin 1% ointment once daily for 5 consecutive days in sufficient quantities to cover affected photodamaged areas where both AKs and SLs were present. The ointment was applied as a thin layer, avoiding application on open wounds or injured skin, as indicated in the package leaflet. Daily sunscreen with a sun protection factor (SPF) of 50 + was recommended. All patients were informed in depth about the possible onset of local skin reactions (LSRs).

Patients were instructed to record the exact timing of onset and the duration of LSRs, if present. They were invited to come to the hospital personally, or to send digital photos when they were unable to attend in person, to allow physicians to evaluate LSRs. LSRs were defined as erythema, scaling, crusting, swelling, vesiculation, or pustulation as well as erosions. The assessment of LSRs was carried out with the use of a semiquantitative 4-point scale with scores of 0—absent, 1—mild (slightly or barely perceptible), 2—moderate (a distinct presence), and 3—severe (marked or intense).

### First Evaluation Post-treatment (T1)

Patients were clinically evaluated 57 days (T1) after the start of the treatment with the acquisition of new clinical images. The lightening effect of tirbanibulin ointment was rated as complete clearance (CC; > 75% improvement up to complete SL disappearance with no evidence of hyperpigmentation), partial clearance (PC; 75–50% reduction in SL pigmentation), or no response (< 50% SL improvement). The efficacy was also assessed according to the SL localization (face or scalp).

For AKs, efficacy was evaluated as CC (total disappearance of the treated lesions), PC (at least 75% reduction), or no response (< 75% reduction). The efficacy was also assessed according to the AK localization (face or scalp).

### Second Evaluation Post-treatment (T2)

Patients who experienced CC of AKs and SLs within the application area at day 57 continued in a 6-month follow-up to assess response durability.

### Statistical Analysis

A descriptive analysis was conducted to evaluate the demographic and clinical characteristics of the patients, stratified by SL and AK clearance. A further descriptive and comparative analysis was performed between patients with and without LSRs.

Considering a non-normal distribution of some of the numerical variables evaluated by the Kolmogorov–Smirnov test for normality, a non-parametric approach was assumed.

The descriptive statistics were reported as medians along with the first and third quartiles (Q_1_–Q_3_) for continuous variables and as absolute frequencies and percentages for categorical variables.

The two-tailed Pearson chi-squared test and the Mann–Whitney *U* test for independent samples were carried out to compare categorical and continuous variables, respectively.

To identify predictors of responsiveness to SL treatment, a multivariate ordinal logistic regression model [7] was performed, including as covariates age, gender, skin phototype, SL position (face or scalp), and LSR onset. The same ordinal logistic regression model was adopted to evaluate the association between AK clearance and the following variables: age, gender, skin phototype, AK position (face or scalp), and the onset of LSRs.

LSRs were categorized as acute (occurring within 24 h) or latent (occurring after 24 h). Onset was defined as the time from the start of drug administration to the beginning of a reaction, and the duration of LSRs was calculated.

Spearman’s rank correlation test was applied to identify associations between SL clearance and AK clearance. For each correlation, the Spearman’s rank correlation coefficient (r_s_) was reported. The same model was used to estimate the associations of LSR onset, severity (absent, mild, moderate, severe), and duration with SL and AK clearance.

Multivariate logistic regression models were performed to identify the factors associated with LSR occurrence. The odds ratios (ORs) with 95% confidence intervals (CIs) were calculated for each covariate of interest in the multivariate models. A *p* value < 0.05 was considered statistically significant. All analyses were performed by using the SPSS statistical package, version 23.0 (IBM Corp., SPSS Statistics, Armonk, NY, USA).

## Results

Forty-two patients (25 male, 17 female) with a median age of 76 years (Q_1_–Q_3_ = 71–80) were enrolled in the study. Half of the patients had a type II skin phototype, while the other half had a type III skin phototype. Most patients had SLs located on the face (*n* = 32, 76.2%), while 23.8% (*n* = 10) had them on the scalp. A similar distribution was also observed for the AKs (face: *n* = 31, 73.8%; scalp: *n* = 11, 26.2%). An average of 6 (4–8) clinically typical, visible, and discrete AKs were treated for each patient. The sociodemographic characteristics of the enrolled patients are shown in Table 1.

**Table 1:** Sociodemographic and clinical characteristics of the patients with SLs and AKs

	Total (*N* = 42)
Age	76 (71–80)
Gender	
Male	25 (59.5%)
Female	17 (40.5%)
Skin phototype	
II	21 (50%)
III	21 (50.0%)
SL location	
Face	10 (23.8%)
Scalp	32 (76.2%)
AK location	
Face	31 (73.8%)
Scalp	11 (26.2%)

**Fig. 1:**
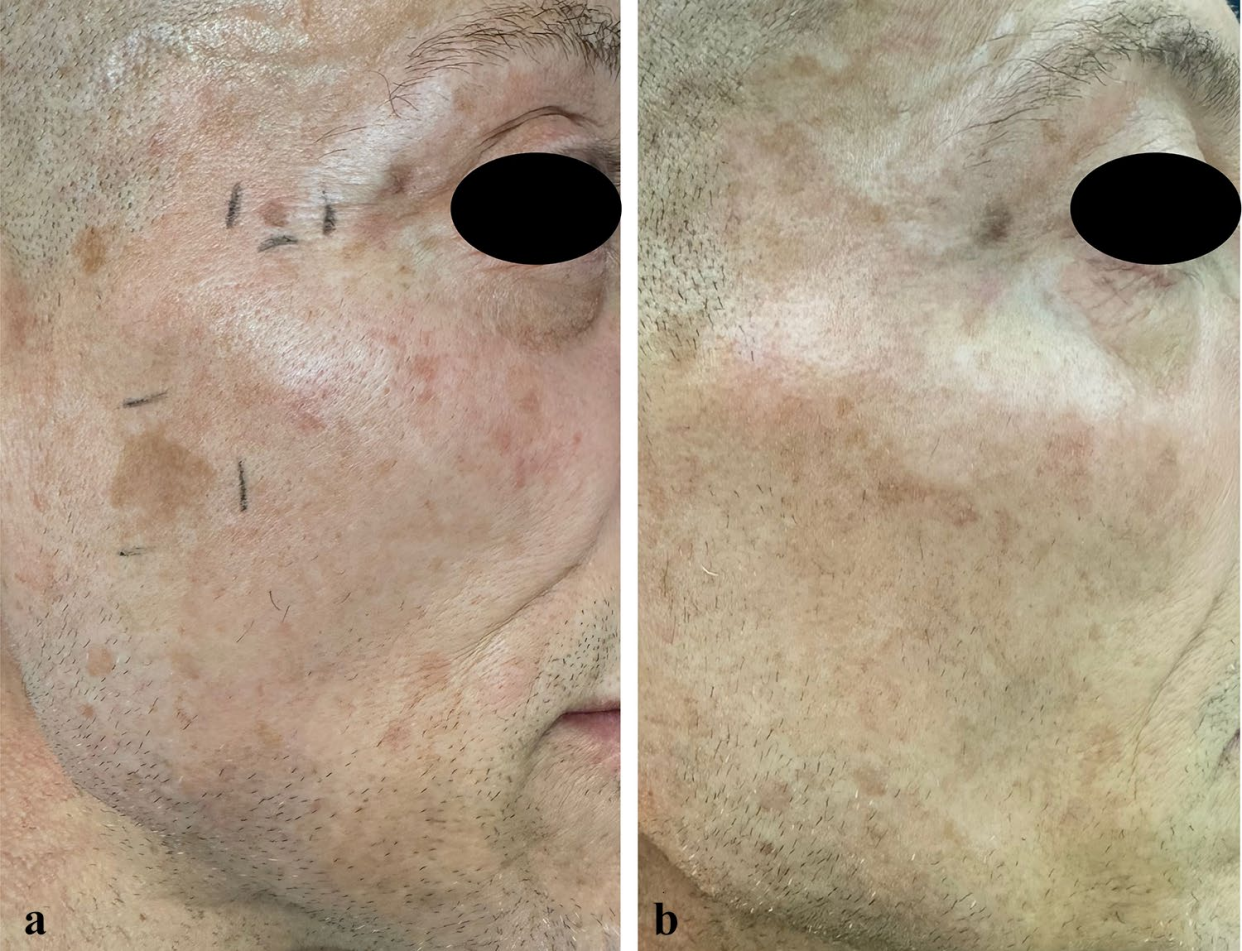
**a** SLs in the right cheek of a 52-year-old man in the context of sun-damaged skin; **b** complete lightening of the treated SLs at the 6-month follow-up

**Fig. 2:**
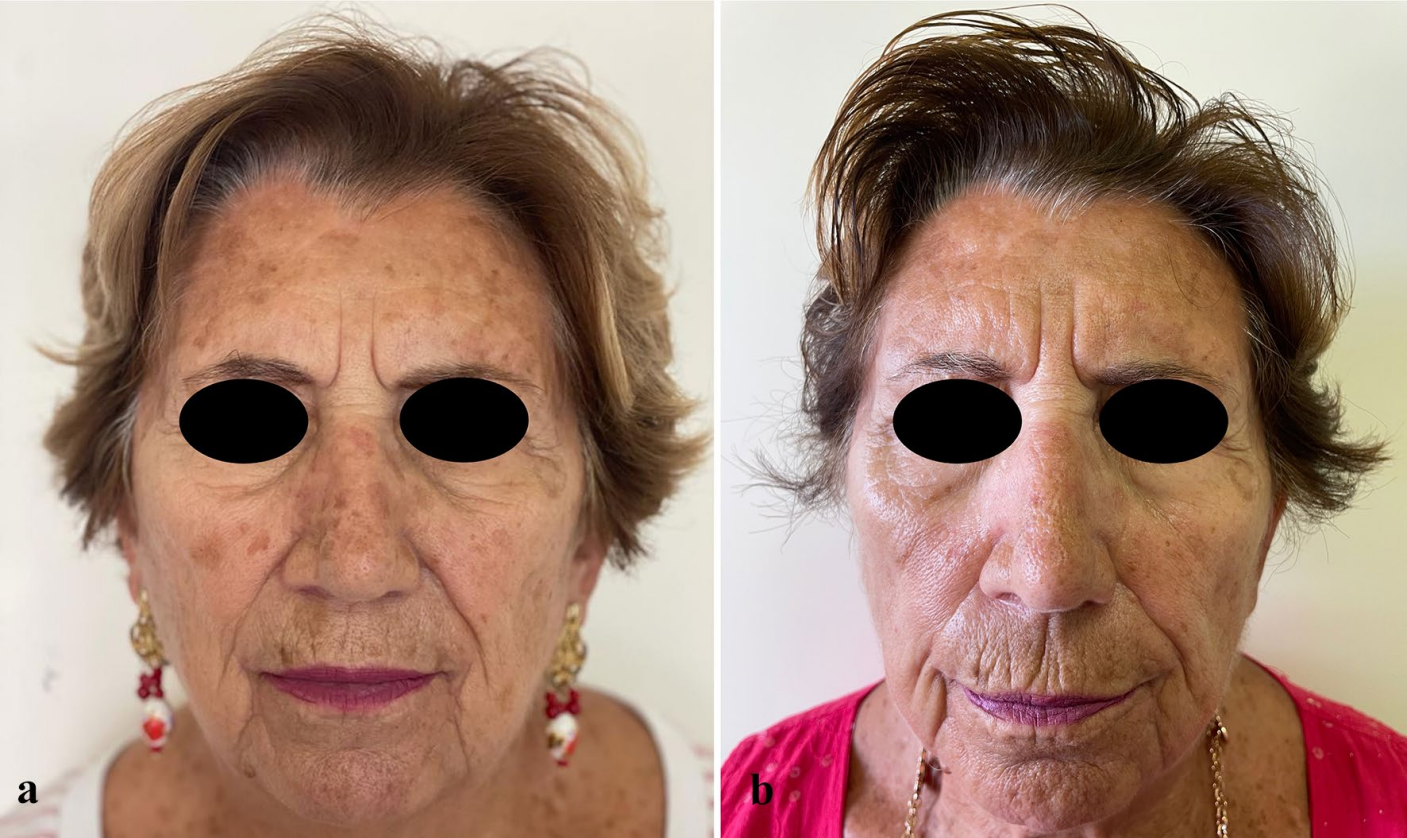
**a** AK in the dorsa of the nose of an 80-year-old woman in the context of photodamaged skin in the cheeks and nose with mottled pigmentation before treatment with tirbanibulin ointment; **b** complete resolution of the AK in the nose and concomitant skin rejuvenation with the clearance of mottled pigmentation in the nose and cheeks and a reduction in undereye wrinkles at the 6-month follow-up

**Fig. 3:**
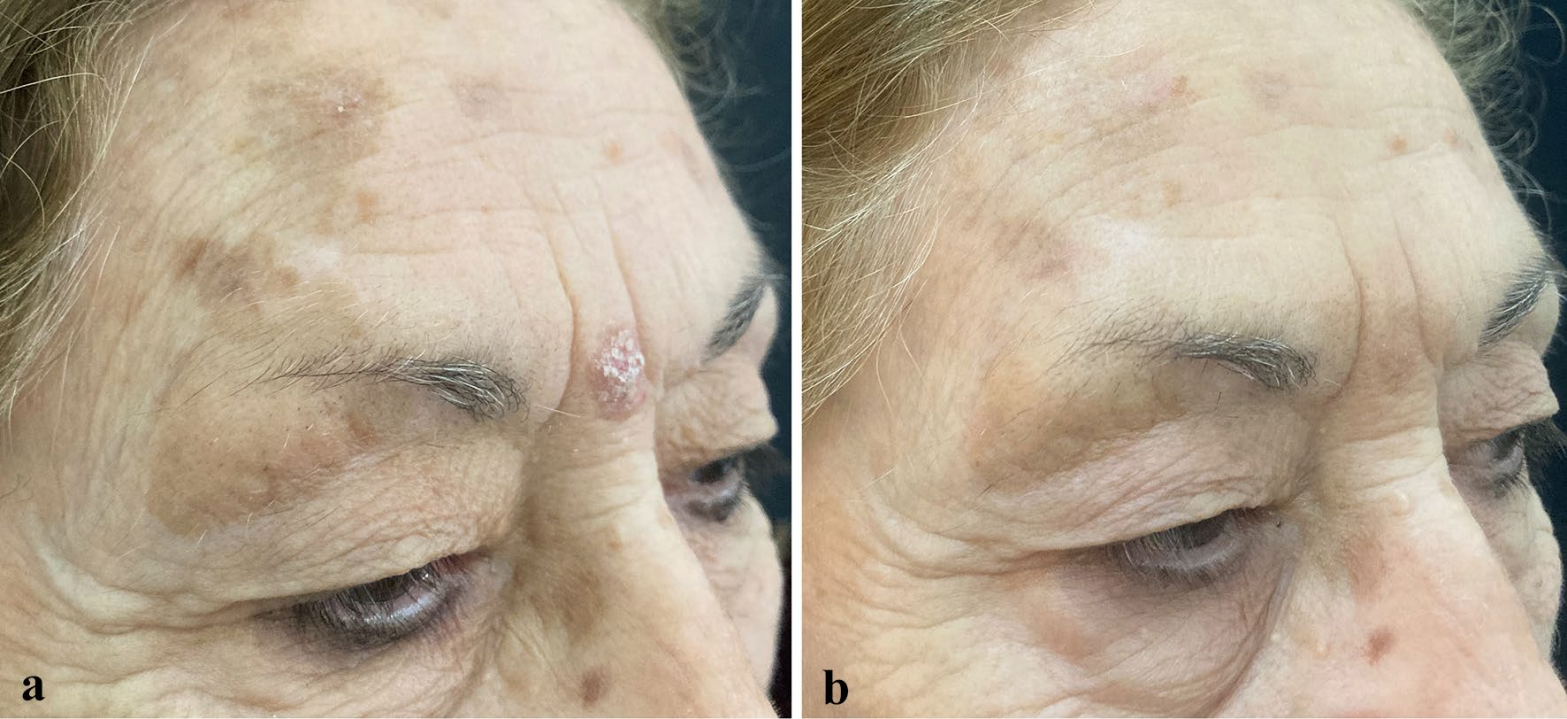
**a** Olsen 3 AK in the glabellar region in the context of sun-damaged skin with large SLs in the right temporal and supraciliary areas; **b** complete AK clearance with significant lightening of the SLs in the right temporal and supraciliary areas at the 6-month follow-up

**Fig. 4:**
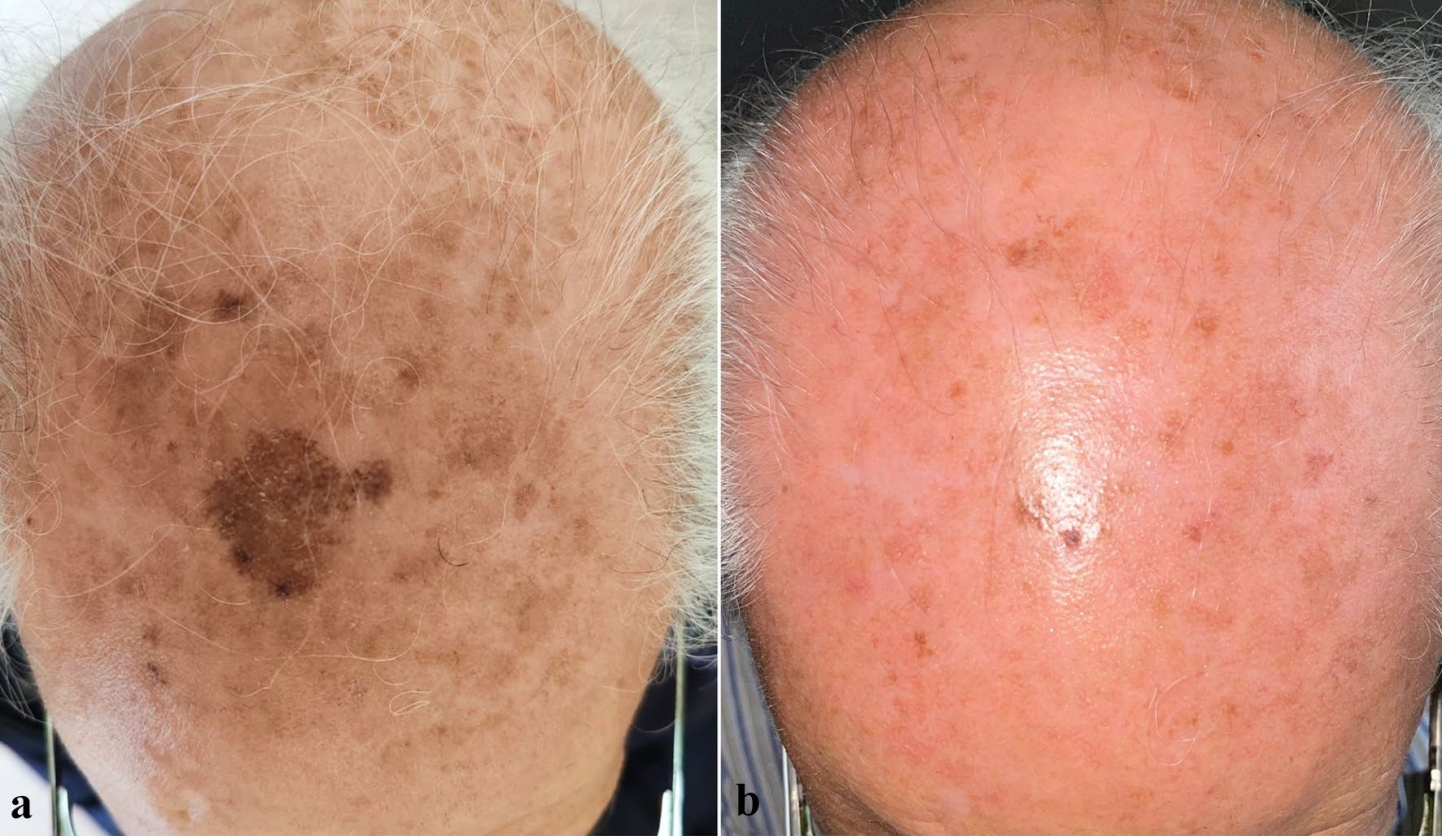
**a** Olsen grade 1 AK lesions of the scalp in the context of sun-damaged skin, with a large pigmented SL, seborrheic keratoses, and diffuse mottled pigmentation; **b** complete resolution of the AKs and the SL at the 6-month follow-up

**Fig. 5:**
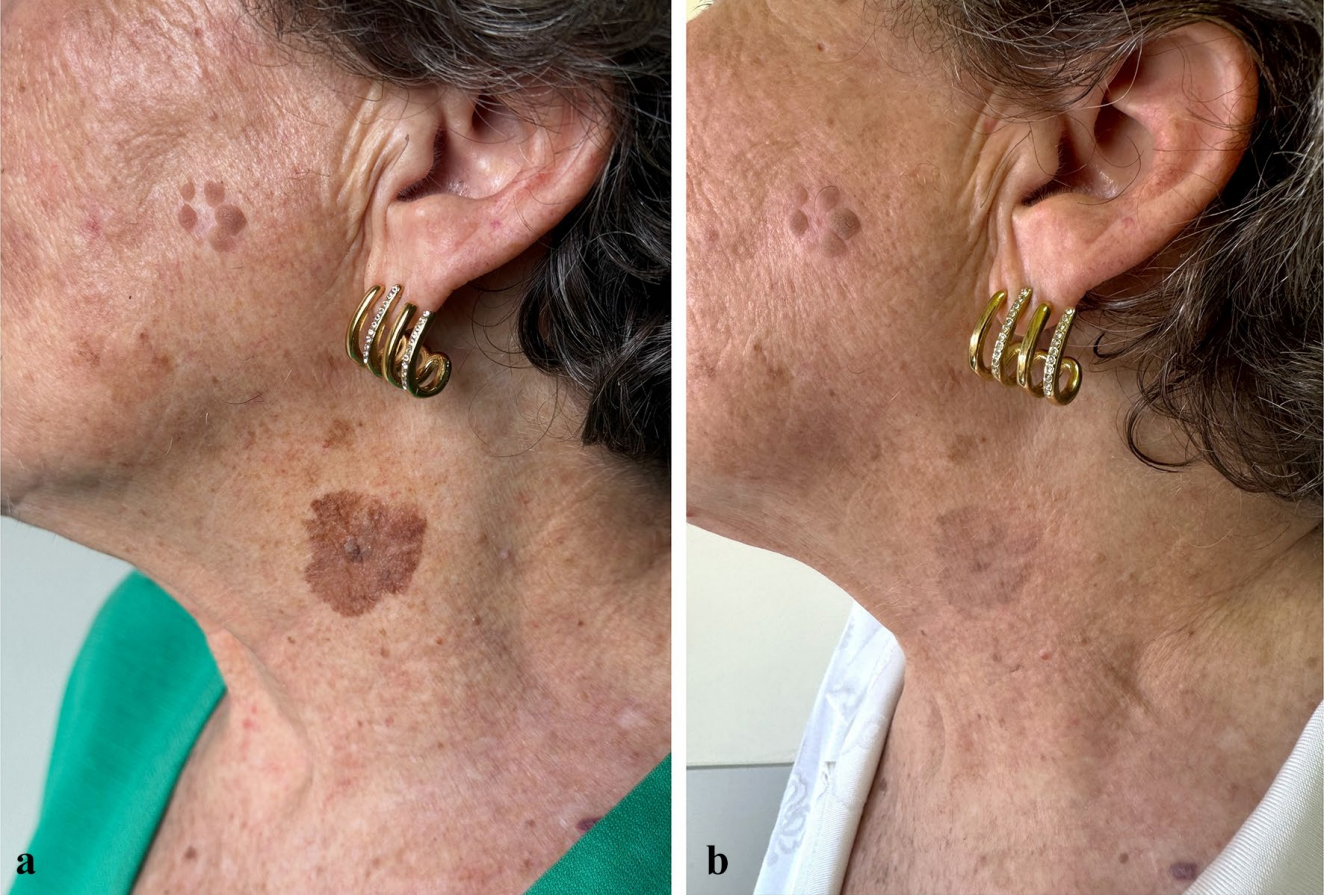
**a** A large SL centered on a seborrheic keratosis in the lateral surface of the neck of a 72-year-old woman before tirbanibulin 1% treatment; **b** partial lightening of the SL and the seborrheic keratosis at the 6-month follow-up after tirbanibulin application

**Fig. 6:**
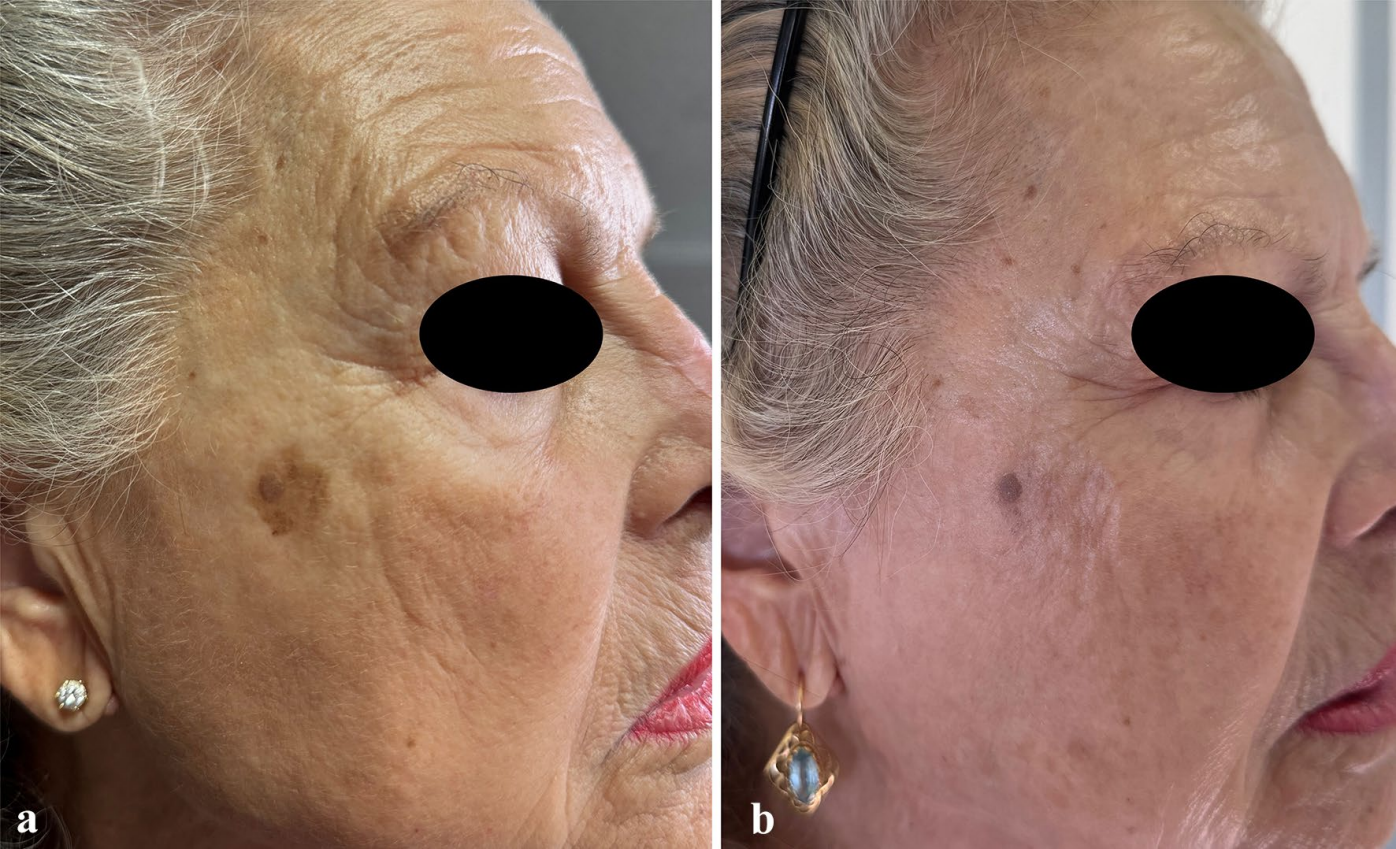
**a** A large SL centered on a tiny seborrheic keratosis in the right cheek of an 82-year-old woman before tirbanibulin 1% treatment; **b** complete SL lightening at the 6-month follow-up after tirbanibulin application

**Fig. 7:**
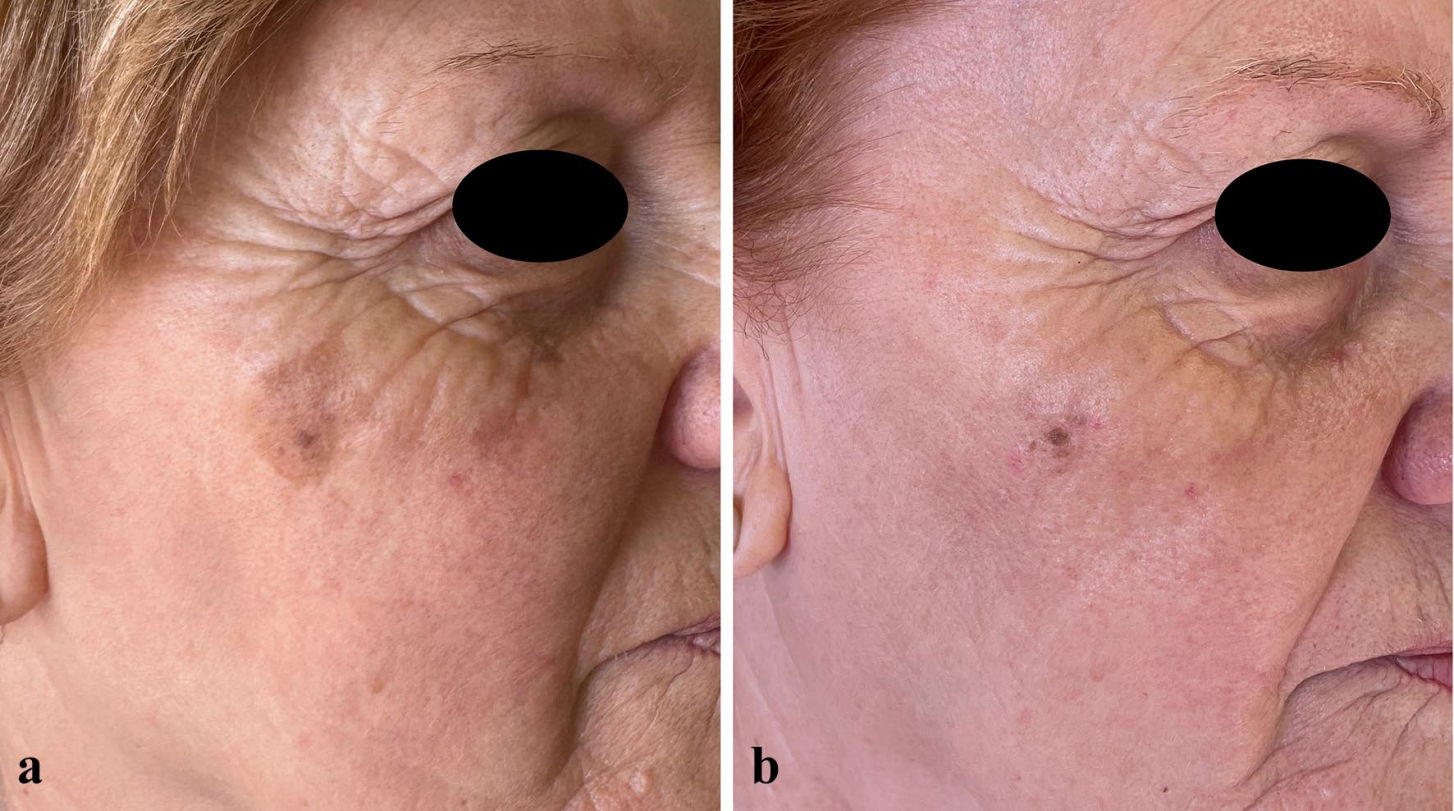
**a** A large SL centered on a tiny seborrheic keratosis in the right cheek of a 71-year-old woman before tirbanibulin 1% treatment; **b** complete SL lightening at the 6-month follow-up after tirbanibulin application

**Fig. 8:**
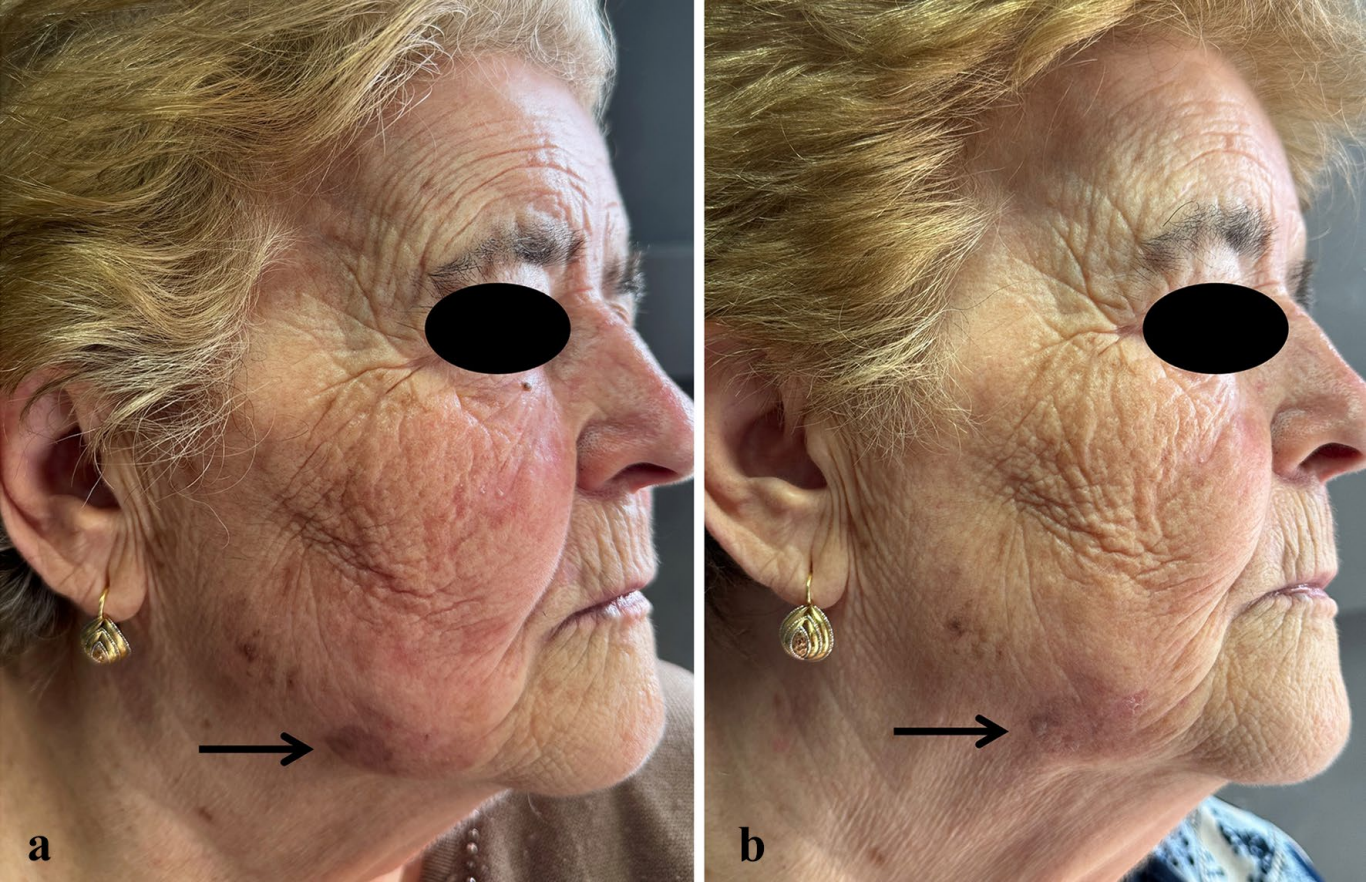
**a** Multiple AKs in the right cheek and a large SL in the homolateral jawline in a 76-year-old woman before starting treatment with tirbanibulin 1% ointment; **b** complete AK clearance and almost total lightening of the SL at the 6-month follow-up

At the 57-day follow-up (T1), CC of SLs was observed in 35.7% of patients (*n* = 15), while 50% of patients (*n* = 21) experienced PC, and 14.3% (*n* = 6) had no response to treatment (Figs. 1, 2, 3, 4, 5, 6, 7, 8; Table 2). No statistically significant difference was observed between the three groups of patients, except for age. No responder patients had a significantly higher median age (median age, Q_1_–Q_3_ = 84, 73–88) compared to those with CC (median age, Q_1_–Q_3_ = 76, 65–80; *p* = 0.036) and those with PC (median age, Q_1_–Q_3_ = 74, 71–80; *p* = 0.036).

**Table 2:** Tirbanibulin efficacy at day 57 in the lightening of SLs

	No. of patients	Complete clearance	Partial clearance	No response
SLs	42	15 (35.7%)	21 (50%)	6 (14.3%)
SL location				
Face	32	12 (37.5%)	16 (50%)	4 (12.5%)
Scalp	10	3 (30%)	5 (50%)	2 (2’%)

Regarding AKs, CC was observed in 52.4% of the patients (*n* = 22), while 40.5% (*n* = 17) had PC; only 7.1% of the patients (*n* = 3) did not respond to treatment (Table 3).

**Table 3:** Tirbanibulin efficacy at day 57 in the treatment of AKs

	No. of patients	Complete clearance	Partial clearance	No response
AKs	42	22 (52.4%)	17 (40.5%)	3 (7.1%)
AK location				
Face	31	16 (52%)	14 (45%)	1 (3%)
Scalp	11	6 (55%)	3 (27%)	2 (18%)

As observed for SLs, age was the only statistically significant difference between the three AK patient groups. No responders had a significantly higher median age than patients with CC (median age, Q_1_–Q_3_: no response vs. CC = 84, 80–84 vs. 75, 68–80; *p* = 0.014). The multivariate ordinal regression analysis revealed a significant direct correlation between female gender and better AK clearance (coefficient: 1.95; 95% CI: 0.26/3.63; *p* value = 0.024). Conversely, no significant correlation was observed between the variables considered and SL clearance (Table 4).

**Table 4:** Multivariate ordinal logistic regression model for SL and AK clearance

	AK efficacy			SL efficacy		
	Coefficient	95% CI	*p* value	Coefficient	95% CI	*p* value
Age	0.06	− 0.02/0.14	0.137	0.06	− 0.02/0.14	0.149
Gender (F)	1.95	0.26/3.63	0.024	0.70	− 0.79/2.20	0.357
Skin phototype (II)	− 0.16	− 1.70/1.37	0.833	0.47	− 0.89/1.83	0.499
SL location (face)	-	-	-	− 0.35	− 1.97/1.27	0.671
LSRs	0.15	− 1.27/1.57	0.833	0.58	− 0.77/1.94	0.398
AK location (face)	0.57	− 1.10/2.28	0.505	-	-	-

**Fig. 9:**
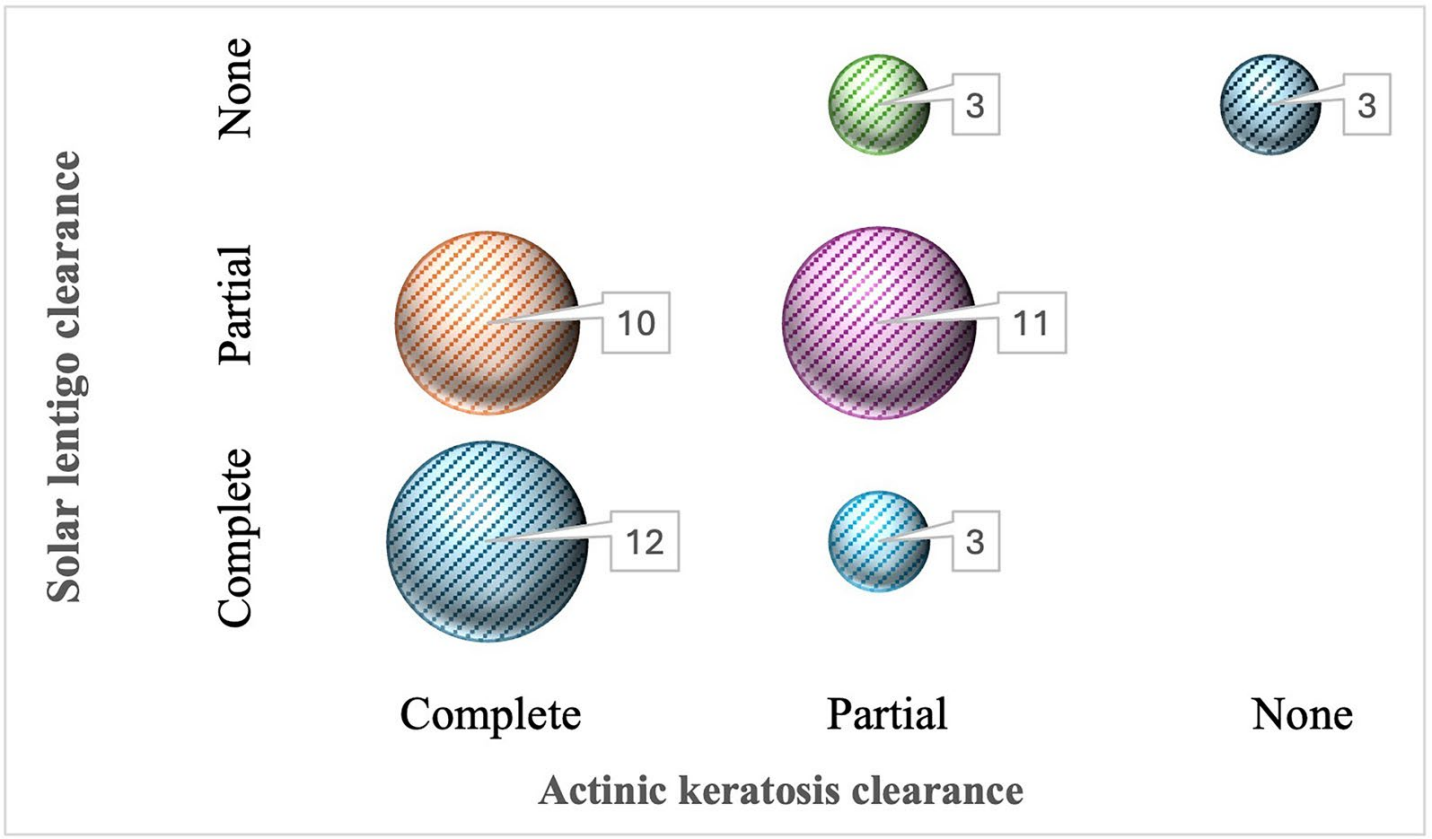
The sample distribution for treatment responses. The size of the bubble is directly related to the sample size (number of patients)

Spearman’s rank correlation analysis revealed a significant positive association between SL and AK clearance (r_s_ = 0.566, *p* < 0.001). Patients who achieved CC of SLs were frequently also complete responders for AKs, and vice versa. Additionally, all of the patients who did not respond to AK treatment were also non-responders for SL. The sample distribution for treatment responses is detailed in the bubble plot shown in Fig. 9.

**Fig. 10:**
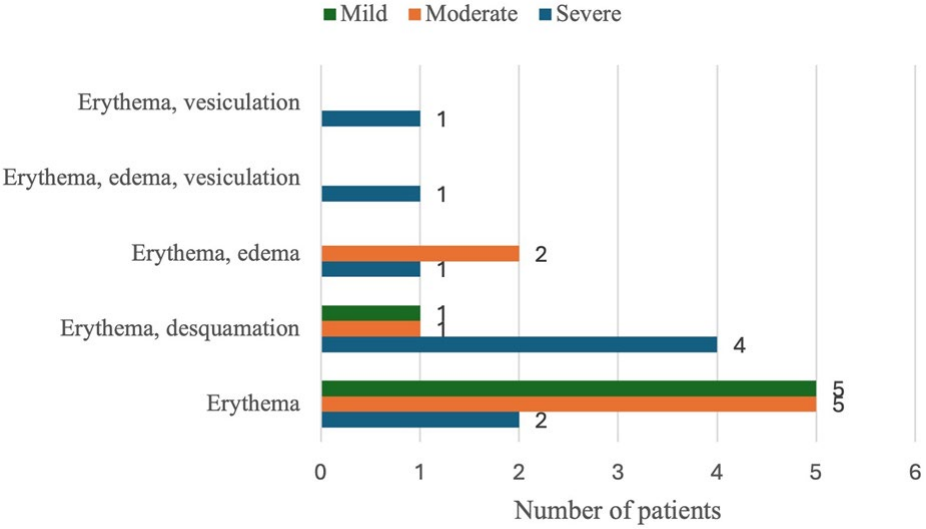
Local skin reactions stratified by severity (none, mild, moderate, severe)

Regarding tirbanibulin tolerability, 54.8% of patients (*n* = 23) developed LSRs, of which 39.8% (*n* = 9/23) were classified as severe, 34.8% (*n* = 8/23) as moderate, and 14.3% (*n* = 6/23) as mild. No patient discontinued the treatment due to the onset of LSRs. Erythema occurred in all patients with LSRs. Approximately half of the severe LSRs caused erythema and desquamation together (Fig. 10). The median duration of the LSRs was 7 days (range 6–9). Six out of 23 patients (26.1%) experienced LSRs acutely, within the first 24 h of therapy, while 17 patients (73.9%) developed LSRs at least 48 h after treatment initiation. Acute onset of LSRs was directly correlated with both SL (r_s_ = 0.449; *p* = 0.032) and AK clearance (r_s_ = 0.375; *p* = 0.078), but these correlations did not reach statistical significance (Table 4). A significantly higher prevalence of skin phototype II was observed in patients with LSRs compared to those without LSRs (65.2% vs. 31.6%; *p* = 0.030). Multivariate logistic regression analysis reported a significantly higher probability of LSRs in patients with skin phototype II compared to III (OR, 95% CI: 0.18, 0.04–0.75; 0.018; Table 5). No patient experienced systemic effects related to the drug.

**Table 5:** Sociodemographic and clinical characteristics of patients stratified by the presence of local skin reactions (LSRs) and factors associated with LSRs

	Without LSRs *N* = 19 (%)	With LSRs *N* = 23 (%)	*p* value	Multivariate OR, 95% CI	*p* value
Age	76 (71–80)	74 (65–80)	0.375	0.92, 0.84–1.01	0.087
Gender (M)				0.55, 0.12–2.46	0.430
Male	14 (73.7	11 (47.8)	0.089		
Female	5 (26.3)	12 (52.2)			
Skin phototype (II)				0.18, 0.04–0.75	0.018
II	6 (31.6)	15 (65.2)	0.030		
III	13 (68.4)	8 (34.8)			
SL location (face)				2.60, 0.42–16.26	0.306
Face	14 (73.7)	18 (78.3)	0.729		
Scalp	5 (26.3)	5 (21.7)			
AK location (face)				1.86, 0.31–11.23	0.499
Face	13 (68.4)	18 (78.3)	0.470		
Scalp	6 (31.6)	5 (21.7)			

At the 6-month follow-up (T2), among patients with CC of SLs (*n* = 15) at T1, two experienced a partial recurrence of hyperpigmentation. Among patients who had CC of AKs (*n* = 22) at T1, 31.8% (*n* = 7) had a recurrence of previously healed lesions.

## Discussion

AKs are pre-cancerous cutaneous lesions resulting from the proliferation of atypical keratinocytes in response to prolonged UV light exposure and commonly arise in the face, scalp, and extremities of fair-skinned patients. AKs arise through a multistep process involving mutations in genes responsible for maintaining epidermal homeostasis, leading to abnormal proliferation of altered keratinocytes and finally resulting in cSCC onset [8, 9]. Being a hallmark of photodamaged skin, AKs are often accompanied by other lesions associated with chronic UV damage, including cutaneous malignancies, such as basal cell carcinoma and melanoma, and benign lesions, such as seborrheic keratoses and SLs [10]. The latter are benign well-demarcated hyperpigmented lesions that arise on chronically photodamaged areas, where the cumulative effect of UV leads to focal keratinocyte hyperplasia and melanocyte proliferation [6, 11]. Despite its benign traits, the potential negative social impact of SLs should not be underrated, since they appear mainly in aesthetically sensitive areas, such as the face and dorsal hands, thus representing a frustrating condition for patients, who often seek treatments to achieve clearer, more youthful-looking skin. However, the outcome can often be disappointing, as many available treatments offer limited and non-permanent results.

Therapeutic options for SLs can be divided into two main categories: physical and topical treatments [12]. Physical ones include laser therapy, cryotherapy, and chemical peels, while topical treatments require the application of active compounds, aiming at inhibiting melanin production, blocking melanocyte stimulation, and suppressing melanin delivery to keratinocytes [13].

A recent systematic review comparing the efficacy rate of SL treatments highlighted that combination therapies are the most effective but are limited by adverse events (AEs) in over two-thirds of patients, while laser-based treatments seem to be the most effective monotherapy option, with a complete response attained in 43% of patients [14]**.** The most used laser source for SL treatment is the Q-switched one, with a 532-nm wavelength that selectively targets melanin pigment [13]. Laser treatment shows a good safety profile, with an AE rate of only 23%; these AEs are mainly represented by pigmentary alterations (hyperpigmentation and hypopigmentation), so this treatment requires caution in darker skin types [13, 14]. Cryotherapy is another widely used treatment for benign hyperpigmented lesions and was once considered the first-line approach for SLs. However, it has shown lower pooled response rates compared with other treatment modalities (a complete response rate of 15%) [14]. Its mechanism of action involves selectively injuring melanocytes through tissue freezing since these cells are particularly sensitive to cold. AEs occur in 33% of patients, with post-inflammatory hyperpigmentation being the most frequent. Chemical peels seem to be the least effective option for SLs [14]**.** Among topical treatments, retinoids are widely used as anti-aging and depigmenting agents due to their ability to accelerate epidermal keratinocyte turnover and desquamation, leading to reduced melanosome transfer and faster melanin loss through the epidermis [15]. However, pooled data showed complete and partial responses in 21% and 45% of patients, respectively [14]. Moreover, topical retinoid use is limited by the so-called “retinoid reaction” that occurs in up to 82% of patients. This reaction, characterized by pruritus, burning, erythema, and peeling, is initiated by proinflammatory cytokines and is due to the free carboxylic acid in the retinoid’s polar end [14].

Hydroquinone, another topical agent that primarily targets tyrosinase, was once considered the gold standard due to its strong anti-pigmenting effects. However, it has associated risks of cytotoxicity and permanent depigmentation that have led to the discontinuation of its use. Arbutin, a hydroquinone derivative, represents a safer alternative, though with lower efficacy. Skin-lightening effects can also come from microbial sources, such as kojic acid, a fungal metabolite that chelates copper to suppress tyrosinase, and azelaic acid, produced by *Pityrosporum ovale*, which competitively inhibits tyrosinase without cytotoxicity. Flavonoids, including aloesin and glabiridin, also exert anti-tyrosinase action while offering antioxidant benefits. In contrast, niacinamide (vitamin B3) reduces hyperpigmentation by inhibiting melanosome transfer from melanocytes to keratinocytes without affecting tyrosinase activity [16]. Finally, tranexamic acid (TXA), commonly used as an antifibrinolytic and procoagulant agent, has demonstrated anti-melanogenesis and anti-inflammatory effects, making it a viable treatment option for hyperpigmentation. While TXA is widely used for melasma and has recently gained approval for indications in some countries, it remains an off-label treatment for various other dyschromias, including SL [17, 18].

The major limitations of the aforementioned treatments are their high economic burden and long-term compliance. They often require multiple sessions over extended periods or daily applications for several months, thus impairing patient adherence. Nevertheless, aesthetic results can be disappointing, since pigmentation may return or the improvements may be minimal. Moreover, some advanced treatments, such as Q-switched lasers, are not readily available in all dermatological centers, making them less accessible for many patients. This combination of high costs, long treatments, and limited access leaves many patients frustrated and highlights the need for more effective, affordable, and widely accessible solutions.

The clearance of SLs in patients treated with topical therapies for AKs and field cancerization has been anecdotally reported in the literature, with only two case reports dealing with the lightening of SLs after the application of imiquimod 3.75% cream [19, 20]. The results of our study confirm, for a larger series of patients, the effectiveness of tirbanibulin 1% ointment in inducing a lightening effect on dark spots, as already observed in our previous experience of 7 patients, besides its well-known positive outcome for AKs.

Tirbanibulin ointment represents the latest-arriving AK treatment, adding to the wide range of therapeutic options already available, including 4% 5-fluorouracil (5-FU) cream, 0.5% 5-FU plus 10% salicylic acid, 3% diclofenac sodium in hyaluronic acid gel, 3.75% and 5% imiquimod cream, and photodynamic therapy [1]. Currently, there are no head-to-head studies comparing tirbanibulin’s efficacy with other topical treatments, likely due to its recent introduction to the market. However, indirect comparisons through a recent meta-analysis indicated that tirbanibulin’s efficacy and safety profile align closely with those of other topical therapies but with a shorter treatment period, which represents its greatest step forward [21].

In our study, at the 57-day follow-up, treatment with tirbanibulin ointment led to CC and PC in 52% and 40% of patients with facial and scalp AKs, respectively, confirming the data from clinical trials and real-life experiences [2, 22–24]. Among patients achieving CC, AK recurrence occurred in 7 out of 22 (31.8%), as expected given the chronic relapsing nature of AKs. In our cohort, tirbanibulin performed better in women and younger patients. We can speculate that this gender difference may be due, at least in part, to thinner skin in women, which may allow for better drug penetration [25]. Conversely, younger patients typically experience faster cell turnover and an enhanced tissue repair capacity, which may amplify the effects of pro-apoptotic and anti-tumor agents such as tirbanibulin, thus promoting the elimination of precancerous cells [26].

Similar positive performances were recorded for SLs, with a CC rate of 35% and a PC rate of 50% at the 2-month follow-up as well as excellent maintenance of results at the 6-month follow-up, with tirbanibulin therefore achieving overall better results than those of much more expensive and time-consuming therapies (i.e., topical retinoids or cryotherapy), being slightly inferior only to laser therapy performances. What sets it apart is its short application regimen of only 5 consecutive days, which provides an effective solution in a much shorter time frame compared to other expensive treatments. Furthermore, tirbanibulin ointment is readily accessible and widely available to all patients, including those living in smaller towns or rural areas. This makes it a practical and inclusive therapeutic option, in contrast to laser-based treatments, thus ensuring that even individuals living far from urban centers can receive effective care.

To date, no studies have explored the depigmenting effect of tirbanibulin. Our study results suggest that tirbanibulin may exert therapeutic effects on both conditions, potentially due to shared molecular mechanisms.

The antineoplastic effects of tirbanibulin 1% ointment are mediated by the inhibition of the proliferation of AK dysplastic keratinocytes through microtubule structure disruption and apoptosis induction. A similar proliferative dysregulation occurs in SLs, where UV radiation leads to keratinocyte hyperplasia and their increased production of melanocyte-activating factors, especially endothelin-1 (EDN-1) and stem cell factor (SCF), which synergistically enhance melanin synthesis [27–29].

Specifically, EDN1 binds to EDN1-B receptors (EDNBR) on melanocytes, activating intracellular pathways such as protein kinase C (PKC) and mitogen-activated protein kinases (MAPK) cascades. Simultaneously, SCF binds to its receptor c-KIT on melanocytes and activates intracellular signaling pathways. The mutual interaction between EDN1/EDNBR and SCF/c-KIT signaling leads to enhanced expression and phosphorylation of microphthalmia-associated transcription factor (MITF), which in turn promotes melanin synthesis and melanocyte proliferation [30, 31]. Tirbanibulin’s direct cytotoxic effect on keratinocytes might impair their ability to produce and secrete EDN-1 and SCF [28, 30, 32]. By inhibiting the EDN-1 and SCF upstream signals that activate MITF, tirbanibulin may indirectly reduce the transcription of key enzymes, thus suppressing melanin production and leading to the skin-lightening effect observed in our patients.

Another potential mechanism of action of tirbanibulin might be the modulation of melanosome transfer from melanocytes to keratinocytes, which is crucial for the distribution of pigment across the epidermis. Microtubules play a significant role in facilitating melanosome transport via melanocyte dendrites.

As tirbanibulin disrupts microtubules, it could be hypothesized that it may impair such transport, thereby reducing cutaneous hyperpigmentation [16, 30, 32, 33]. Such a hypothesis may, at least in part, explain how, in our study, the patients who had an excellent response to AKs simultaneously also had complete clearance of SLs.

Our study has some limitations, including the small sample size and the absence of a control group. Additionally, the reduction of SLs was assessed on clinical criteria without instrumental evaluations. Seasonal variations in sunlight exposure during treatment administration represent a potential source of bias, as they possibly influenced biological responses and introduced variability among patients. Furthermore, the durability of the results should be assessed over a longer follow-up period to observe potential clinical recurrences. Finally, the mechanisms by which tirbanibulin exerts its skin-lightening effects on SLs need to be investigated in depth.

## Conclusions

Our study highlighted the dual effect of tirbanibulin 1% ointment in improving the appearance of photoaged skin at application sites used for treating AKs. In a stunning observation, the complete clearance of SLs was observed in 35% of patients, an outcome not reported after any other AK therapies. Further research is essential to elucidate the underlying mechanisms that drive these cosmetic improvements and potentially allow patients to treat these aesthetically bothersome lesions in a few days at limited cost.

## Data Availability

Data are contained within the article.

## References

[1] Eisen DB, Asgari MM, Bennett DD, Connolly SM, Dellavalle RP, Freeman EE, Goldenberg G, Leffell DJ, Peschin S, Sligh JE, et al. Guidelines of care for the management of actinic keratosis. J Am Acad Dermatol. 2021;85:e209–33. 10.1016/j.jaad.2021.02.082. 33820677 10.1016/j.jaad.2021.02.082 PMC12255292

[2] Blauvelt A, Kempers S, Lain E, Schlesinger T, Tyring S, Forman S, Ablon G, Martin G, Wang H, Cutler DL, et al. Phase 3 trials of tirbanibulin ointment for actinic keratosis. N Engl J Med. 2021;384:512–20. 10.1056/NEJMoa2024040. 33567191 10.1056/NEJMoa2024040

[3] Gilaberte Y, Fernández-Figueras MT. Tirbanibulina: revisión de su mecanismo de acción novedoso y de cómo encaja en el tratamiento de la queratosis actínica. Actas Dermosifiliogr. 2022;113:58–66. 10.1016/j.ad.2021.07.006. 10.1016/j.ad.2021.07.006 35249711

[4] Fidanzi C, Bevilacqua M, Salvia G, Romanelli M, Janowska A. Tirbanibulin and solar lentigo clearance: an aesthetically pleasing side effect? Int J Dermatol. 2024;63:528–9. 10.1111/ijd.17054. 10.1111/ijd.17054 38305473

[5] Li Pomi F, Peterle L, d’Aloja A, Di Tano A, Vaccaro M, Borgia F. Anti-aging effects of tirbanibulin 1% ointment: a real-life experience. Dermatol Ther (Heidelb). 2024;14:1683–96. 10.1007/s13555-024-01178-0. 10.1007/s13555-024-01178-0 PMC11169325 38740726

[6] Choi W, Yin L, Smuda C, Batzer J, Hearing VJ, Kolbe L. Molecular and histological characterization of age spots. Exp Dermatol. 2017;26:242–8. 10.1111/exd.13203. 27621222 10.1111/exd.13203 PMC5342934

[7] Ananth C. Regression models for ordinal responses: a review of methods and applications. Int J Epidemiol. 1997;26:1323–33. 10.1093/ije/26.6.1323. 9447413 10.1093/ije/26.6.1323

[8] Fania L, Didona D, Di Pietro FR, Verkhovskaia S, Morese R, Paolino G, Donati M, Ricci F, Coco V, Ricci F, et al. Cutaneous squamous cell carcinoma: from pathophysiology to novel therapeutic approaches. Biomedicines. 2021;9:171. 10.3390/biomedicines9020171. 33572373 10.3390/biomedicines9020171 PMC7916193

[9] Papa V, Li Pomi F, Borgia F, Vaccaro M, Pioggia G, Gangemi S. Immunosenescence and skin: a state of art of its etiopathogenetic role and crucial watershed for systemic implications. Int J Mol Sci. 2023;24:7956. 10.3390/ijms24097956. 37175661 10.3390/ijms24097956 PMC10178319

[10] Del Bino S, Duval C, Bernerd F. Clinical and biological characterization of skin pigmentation diversity and its consequences on UV impact. Int J Mol Sci. 2018;19:2668. 10.3390/ijms19092668. 30205563 10.3390/ijms19092668 PMC6163216

[11] Warrick E, Duval C, Nouveau S, Bastien P, Piffaut V, Chalmond B, Ortonne J-P, de Lacharrière O, Bernerd F. Morphological and molecular characterization of actinic lentigos reveals alterations of the dermal extracellular matrix. Br J Dermatol. 2017;177:1619–32. 10.1111/bjd.15697. 28570000 10.1111/bjd.15697

[12] Ortonne J-P, Pandya AG, Lui H, Hexsel D. Treatment of solar lentigines. J Am Acad Dermatol. 2006;54:S262–71. 10.1016/j.jaad.2005.12.043. 16631967 10.1016/j.jaad.2005.12.043

[13] Passeron T, Genedy R, Salah L, Fusade T, Kositratna G, Laubach H-J, Marini L, Badawi A. Laser treatment of hyperpigmented lesions: position statement of the European Society of Laser in Dermatology. J Eur Acad Dermatol Venereol. 2019;33:987–1005. 10.1111/jdv.15497. 10.1111/jdv.15497 30873649

[14] Mukovozov I, Roesler J, Kashetsky N, Gregory A. Treatment of lentigines: a systematic review. Dermatol Surg. 2023;49:17–24. 10.1097/DSS.0000000000003630. 36533790 10.1097/DSS.0000000000003630

[15] Ebanks JP, Wickett RR, Boissy RE. Mechanisms regulating skin pigmentation: the rise and fall of complexion coloration. Int J Mol Sci. 2009;10:4066–87. 10.3390/ijms10094066. 19865532 10.3390/ijms10094066 PMC2769151

[16] Gillbro JM, Olsson MJ. The melanogenesis and mechanisms of skin-lightening agents—existing and new approaches. Int J Cosmet Sci. 2011;33:210–21. 10.1111/j.1468-2494.2010.00616.x. 21265866 10.1111/j.1468-2494.2010.00616.x

[17] Chen T, Xue J, Wang Q. Tranexamic acid for the treatment of hyperpigmentation and telangiectatic disorders other than melasma: an update. Clin Cosmet Investig Dermatol. 2024;17:2151–63. 10.2147/CCID.S479411. 10.2147/CCID.S479411 PMC11439988 39350932

[18] Kim KM, Lim HW. The uses of tranexamic acid in dermatology: a review. Int J Dermatol. 2023;62:589–98. 10.1111/ijd.16160. 35323992 10.1111/ijd.16160

[19] Cantisani C, Cantoresi F, Mercuri SR, Binic I, Golubovic M, Marino R, Paolino G. Efficacy of Imiquimod 3.75% cream for the treatment of solar lentigo. Dermatol Ther. 2020. 10.1111/dth.13256. 32031733 10.1111/dth.13256

[20] Di Bartolomeo L, Guarneri F, Moretti G. Treatment of solar lentigo with imiquimod 3.75% cream: a dermoscopic study. J Cosmet Dermatol. 2022;21:6487–9. 10.1111/jocd.15177. 35748519 10.1111/jocd.15177

[21] Heppt MV, Dykukha I, Graziadio S, Salido-Vallejo R, Chapman-Rounds M, Edwards M. Comparative efficacy and safety of tirbanibulin for actinic keratosis of the face and scalp in Europe: a systematic review and network meta-analysis of randomized controlled trials. J Clin Med. 2022;11:1654. 10.3390/jcm11061654. 35329979 10.3390/jcm11061654 PMC8952421

[22] Kirchberger MC, Gfesser M, Erdmann M, Schliep S, Berking C, Heppt MV. Tirbanibulin 1% ointment significantly reduces the actinic keratosis area and severity index in patients with actinic keratosis: results from a real-world study. J Clin Med. 2023;12:4837. 10.3390/jcm12144837. 37510952 10.3390/jcm12144837 PMC10381110

[23] Campione E, Rivieccio A, Gaeta Shumak R, Costanza G, Cosio T, Lambiase S, Garofalo V, Artosi F, Lozzi F, Freni C, et al. Preliminary evidence of efficacy, safety, and treatment satisfaction with tirbanibulin 1% ointment: a clinical perspective on actinic keratoses. Pharmaceuticals. 2023;16:1686. 10.3390/ph16121686. 10.3390/ph16121686 PMC10748142 38139813

[24] Li Pomi F, Vaccaro M, Pallio G, Rottura M, Irrera N, Borgia F. Tirbanibulin 1% ointment for actinic keratosis: results from a real-life study. Medicina (Buenos Aires). 2024;60:225. 10.3390/medicina60020225. 10.3390/medicina60020225 PMC10890708 38399512

[25] Giacomoni PU, Mammone T, Teri M. Gender-linked differences in human skin. J Dermatol Sci. 2009;55:144–9. 10.1016/j.jdermsci.2009.06.001. 19574028 10.1016/j.jdermsci.2009.06.001

[26] Khalid KA, Nawi AFM, Zulkifli N, Barkat MdA, Hadi H. Aging and wound healing of the skin: a review of clinical and pathophysiological hallmarks. Life. 2022;12:2142. 10.3390/life12122142. 36556508 10.3390/life12122142 PMC9784880

[27] Takenaka Y, Hoshino Y, Nakajima H, Hayashi N, Kawashima M, Imokawa G. Paracrine cytokine mechanisms underlying the hyperpigmentation of seborrheic keratosis in covered skin areas. J Dermatol. 2013;40:533–42. 10.1111/1346-8138.12178. 23662587 10.1111/1346-8138.12178

[28] Kadono S, Manaka I, Kawashima M, Kobayashi T, Imokawa G. The role of the epidermal endothelin cascade in the hyperpigmentation mechanism of lentigo senilis. J Investig Dermatol. 2001;116:571–7. 10.1046/j.1523-1747.2001.01296.x. 10.1046/j.1523-1747.2001.01296.x 11286625

[29] Hattori H, Kawashima M, Ichikawa Y, Imokawa G. The epidermal stem cell factor is over-expressed in lentigo senilis: implication for the mechanism of hyperpigmentation. J Investig Dermatol. 2004;122:1256–65. 10.1111/j.0022-202X.2004.22503.x. 10.1111/j.0022-202X.2004.22503.x 15140230

[30] Goorochurn R, Viennet C, Granger C, Fanian F, Varin-Blank N, Le Roy C, Humbert P. Biological processes in solar lentigo: insights brought by experimental models. Exp Dermatol. 2016;25:174–7. 10.1111/exd.12937. 26739821 10.1111/exd.12937

[31] Upadhyay PR, Ho T, Abdel-Malek ZA. Participation of keratinocyte- and fibroblast-derived factors in melanocyte homeostasis, the response to UV, and pigmentary disorders. Pigment Cell Melanoma Res. 2021;34:762–76. 10.1111/pcmr.12985. 33973367 10.1111/pcmr.12985 PMC8906239

[32] Imokawa G. Melanocyte activation mechanisms and rational therapeutic treatments of solar lentigos. Int J Mol Sci. 2019;20:3666. 10.3390/ijms20153666. 31357457 10.3390/ijms20153666 PMC6695993

[33] Schlesinger T, Stockfleth E, Grada A, Berman B. Tirbanibulin for actinic keratosis: insights into the mechanism of action. Clin Cosmet Investig Dermatol. 2022;15:2495–506. 10.2147/CCID.S374122. 36415541 10.2147/CCID.S374122 PMC9675993

